# Using *de novo* protein structure predictions to measure the quality of very large multiple sequence alignments

**DOI:** 10.1093/bioinformatics/btv592

**Published:** 2015-11-14

**Authors:** Gearóid Fox, Fabian Sievers, Desmond G. Higgins

**Affiliations:** Conway Institute of Biomolecular and Biomedical Research, and UCD School of Medicine and Medical Science, University College Dublin, Dublin 4, Ireland

## Abstract

**Motivation:** Multiple sequence alignments (MSAs) with large numbers of sequences are now commonplace. However, current multiple alignment benchmarks are ill-suited for testing these types of alignments, as test cases either contain a very small number of sequences or are based purely on simulation rather than empirical data.

**Results:** We take advantage of recent developments in protein structure prediction methods to create a benchmark (ContTest) for protein MSAs containing many thousands of sequences in each test case and which is based on empirical biological data. We rank popular MSA methods using this benchmark and verify a recent result showing that chained guide trees increase the accuracy of progressive alignment packages on datasets with thousands of proteins.

**Availability and implementation:** Benchmark data and scripts are available for download at http://www.bioinf.ucd.ie/download/ContTest.tar.gz.

**Contact:** des.higgins@ucd.ie

**Supplementary information:** Supplementary data are available at *Bioinformatics* online.

## 1 Introduction

Making a multiple sequence alignment (MSA) of nucleotide or amino acid sequences is a crucial step needed in a wide variety of bioinformatics studies. To overcome the extreme computational demands of exact algorithms, MSA software uses heuristics to find near-optimal alignments in a reasonable time. Different software packages, however, use different heuristics and objective functions and explicitly focus on different aspects of the MSA process such as accurate placement of gaps for phylogenetic reconstruction of small numbers of sequences (Löytynoja and Goldman, 2005) or sheer speed for making very large alignments e.g. MAFFT PartTree (Katoh and Toh, 2007). Accurate benchmarks are therefore important to allow users of MSA software to choose the most appropriate tools and to guide developers in improving algorithms and heuristics (Iantorno *etal.*, 2014).

As computational resources and sequence databases grow, and methods for creating MSAs are improved, the practical upper limit for the size of MSAs increases. Currently, alignments of tens of thousands of sequences can be made routinely on a desktop computer. It is therefore important that benchmarks can reflect alignment problems on this scale. Current protein MSA benchmarks can be divided into structure-, phylogeny-, simulation- and consistency-based benchmarks (Iantorno *etal.*, 2014). Structure- and phylogeny-based benchmarks, in which scores are based on structural superpositions and accurate inference of phylogenetic trees, respectively, are strongly grounded in empirical biological data, but they focus on alignments of small numbers of sequences and are difficult to scale to larger datasets. Meanwhile, simulation- and consistency-based benchmarks are based on simulations of protein evolution and simple agreement between different MSA methods, respectively, and can involve alignments of arbitrary size. It is unclear, however, how well simulated sequences model actual biological sequences, while consistency measures only how similar the results of one heuristic method are to the results of other heuristic methods.

MSAs containing large numbers of sequences are generally created by software using the progressive alignment method. Progressive alignment approximates the optimal alignment of multiple sequences by calculating a series of pairwise alignments according to a bifurcating tree known as a guide tree (Higgins *etal.*, 1992). Recently, completely imbalanced—or ‘chained’—guide trees have been shown to increase the accuracy of some progressive MSA methods (Boyce *etal.*, 2014) when aligning large numbers of sequences. This result is controversial (Boyce *etal.*, 2015; Tan *etal.*, 2015) and a benchmark is needed to accurately test the effect of chained guide trees on alignment accuracy.

**Fig. 1.**
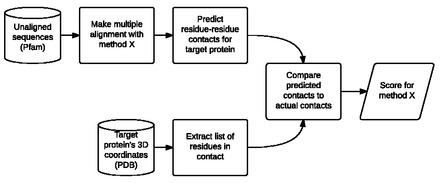
Flowchart of benchmark process for one test case. Sequences from Pfam are aligned with the method of interest and the resulting MSA is used to predict residue–residue contacts for one of the proteins in the alignment. The 3D coordinates of this target protein are used to calculate the true residue–residue contacts. The two lists of contacts are compared to calculate a score for the alignment

Here, we describe a benchmark, ContTest, that is able to realistically test the accuracy of MSAs of very large numbers of amino acid sequences, making use of recent developments in the prediction of protein structures from large MSAs (Marks *etal.*, 2011). We take the MSA that is to be tested and use it to predict a contact map for a protein in the alignment that also has a known three-dimensional (3D) structure. This predicted contact map is then compared with the known contact map for the same protein, and the alignment is scored based on their agreement (Fig. 1). The benchmark makes use of all the sequences in the alignment and does not reward over-alignment. It also avoids superposition of predicted and known structures as this step itself requires the use of heuristic algorithms. This process gives a very robust scoring system based on the assumption that, from a protein structure perspective, better alignment methods will result in more accurate contact maps.

We used the ContTest benchmark to compare the accuracy of some widely used MSA packages. We also used it to compare some different ways of using these packages, e.g. by varying the numbers of iterations and in particular using chained guide trees with the progressive alignment methods.

### 1.1 De novo contact prediction

Recently, several computationally efficient methods have been developed to predict protein structural information solely from an alignment of the sequence of interest with a large number of homologous sequences (Jones *etal.*, 2012; Marks *etal.*, 2011, 2012; Taylor *etal.*, 2013). Residues that are in contact in the folded protein can exhibit a pattern of coevolution, where substitutions at one position may be compensated for by complementary mutations at nearby positions. EVfold-mfDCA (Marks *etal.*, 2011) and PSICOV (Jones *etal.*, 2012) detect correlations between the patterns of substitutions in pairs of columns of an MSA and output a list of pairs of residues predicted to be in contact.

We investigated if we could use the accuracy of contact predictions to infer the accuracy of the input MSAs. For a target protein with a resolved 3D structure in the Protein Data Bank (PDB) (Berman *etal.*, 2000) and a large number of homologous sequences in the Pfam database (Finn *etal.*, 2014), we compare contact predictions made using PSICOV or EVfold-mfDCA from an alignment of the sequences to a list of known contacts derived from the protein’s 3D coordinates. Accuracy is evaluated as the precision of the top L/5 long range contacts predicted, where *L* is the length of the target protein. Long range is defined to mean any contact between residues separated by at least 23 other residues.

## 2 Materials and methods

### 2.1 Software versions and parameters

We used the following software to make MSAs:

- Clustal Omega 1.2.0 (Sievers *etal.*, 2011). For one, two or three iterations we use the parameters:
  clustalo -i … --guidetree-out=… -o …

where the output of one iteration is reused as the input file to the next iteration. To use an external HMM we use the parameters:

clustalo -i … --hmm-in=… -o …

‘Chained’ guide trees are generated internally using the –pileup option:

clustalo --pileup -i … -o …

- Clustal W2.1 (Larkin *et al.*, 2007). We reuse the guide trees created by Clustal Omega:

clustalw2 -quiet -infile=… -outfile=…

-usetree=… -outorder=input

We convert the output file from Clustal format to FASTA format using the sreformat utility from HMMER.

hmmt -o … hmm_out infile

kalign-q-i…-o…

kalign -f -q -i … -o …

- HMMER 1.8.5 (hmmt) (Eddy, 1998)

- Kalign 2.04 (Lassmann *et al.*, 2009)

- Kalign 1.04 (Lassmann and Sonnhammer, 2005)

- MAFFT v7.029b (Katoh and Standley, 2013) Default (FFT-NS-2):
  mafft --anysymbol in > out

To make alignments using ‘chained’ guide trees, we create an external guide tree file in MAFFT tree format, which is read using the --treein option:

mafft --anysymbol --treein … in > out

NW-NS-PartTree-1:

mafft --retree 1 --maxiterate 0 --nofft

--parttree --anysymbol in > out

- MUSCLE v3.8.31 (Edgar, 2004). One iteration:
  muscle -maxiters 1 -diags1 -sv
  -distance kbit20_3 -in … -out …

Two iterations:

muscle -maxiters 2 -in … -out …

To make alignments using ‘chained’ guide trees, we create an external guide tree file in Newick tree format, which is read using the -usetree option. One iteration, chained guide tree: muscle -maxiters 1 -diags1 -sv -usetree …

-distance kbit20_3 -in … -out …

Two iterations, chained guide tree:

muscle -usetree … -maxiters 2 -in …

-out …

We used PSICOV version 2.1 (Jones *etal.*, 2012) and FreeContact version 1.0.21 (Kajan *etal.*, 2014) to predict residue–residue contacts from MSAs. We used PSICOV with default parameters and FreeContact with the --parprofevfold option.

We used CMView 1.1.1 (Vehlow *etal.*, 2011) to extract contact maps from PDB files.

### 2.2 Test case selection and preparation

We based our test cases on the 150 proteins used to test contact prediction software by Jones *et al.* (2012). Each test case consists of the full set of sequences from a single Pfam family and a list of experimentally determined residue–residue contacts for a protein in that family. We retrieved 3D co-ordinates for each target protein from the PDB and extracted the list of long range residue–residue contacts using CMview (Vehlow *etal.*, 2011), using an 8 Ångström $C_{\beta }$–$C_{\beta }$ distance threshold ($C_{\alpha }$ for glycine) and a minimum sequence separation of 24 positions. We identified Pfam families for each target protein and downloaded sequences from Pfam in FASTA format, with all gaps represented by ‘–’ characters and all characters uppercase. Sequence orders were randomly shuffled before making alignments. Where the sequence of a protein differs between the PDB and Pfam, we perform a pairwise alignment between the PDB sequence and the Pfam sequence to correctly map columns of the alignment to beta carbons in the known structure.

Test cases were excluded where PSICOV was unable to make predictions from any of the alignments tested due to the alignments containing too few sequences. Test cases were also excluded where there were too many sequences in the Pfam family to be aligned by one or more of the alignment packages tested. For instance, Clustal W2 is unable to align more than 40 000 sequences, and Kalign 2 is unable to align more than 60 000 sequences.) One test case had too few true positive contacts to be useful for the benchmark. Fourteen test cases were excluded using these criteria, leaving a total of 136 test cases for the benchmark. Details of the test cases are given in Supplementary Table S1.

### 2.3 Scoring predicted contacts

The PSICOV and Evfold-mfDCA algorithms both assign scores to each predicted contact. We assess the quality of long range contact predictions; for amino acid residues at positions *i* and *j* in the protein chain, a contact is defined as long range if |j−i|>23. For a test case with a target protein of length *L*, we calculate the precision of the top scoring L/5 long range contacts output by PSICOV and EVfold-mfDCA, which we refer to as the PSICOV precision and the EVfold-mfDCA precision for that test case, respectively.

Because of the dependence of the output MSA on input sequence order for most methods tested, where possible we made three alignments for each test case with each alignment method, randomizing the order of the input sequences before each alignment. We calculated the average PSICOV precision and average EVfold-mfDCA precision over the three replicates. Then, we calculated the geometric mean of the average PSICOV and average EVfold-mfDCA precisions to arrive at a single number representing the precision of contact predictions for that test case. We call this the ContTest score for that test case. We rank alignment methods by the mean ContTest score over all test cases. Pfam stores only a single alignment for each family, while Clustal Omega runs extremely slowly when using chained guide trees. Therefore, results for Pfam and Clustal Omega (chained guide tree) are based on a single replicate only.

While this scoring method involves only a subset of columns in the alignment, we found that using receiver-operating characteristic curves to score predicted contacts produced almost the same rankings of alignments (Supplementary Text and Supplementary Data and Supplementary Data).

### 2.4 Statistical significance of benchmark scores

We calculate the statistical significance of the difference in two benchmark scores by using the Wilcoxon signed rank test where the ContTest scores for each of the 136 test cases are paired between the two conditions.

### 2.5 Making ‘bad’ alignments

To validate our benchmark, we use two methods to artificially create alignments which we expect to be worse than some reference alignment. First, given a reasonable starting alignment of a set of sequences, we expect that shifting a random subset of the sequences out of alignment with the others will result in an alignment that is poorer than the original alignment. We shift sequences out of alignment by adding a gap character to the start of the aligned sequence and truncating the last character. For each test case, we take the Pfam alignment and shift 0.5%, 1%, 2%, 3%, 4% or 5% of the sequences out of alignment, creating a series of alignments which we expect to be of decreasing quality and which should therefore result in decreasing benchmark scores.

Second, we perform a similar analysis by mutating random residues, which we expect should decrease contact prediction accuracy. Starting with the Pfam alignments, we mutate between 0 and 20% of residues in each alignment to a different residue. We expect that increasing numbers of mutations should again result in a decreasing ContTest score.

### 2.6 Adding HOMSTRAD reference sequences to ContTest alignments

We found mappings from 80 of the ContTest test cases to HOMSTRAD (Mizuguchi *etal.*, 1998) alignments using the PFAM.db package for R (Carlson *etal.*, n.d.). For each of these 80 test cases, we added the dealigned HOMSTRAD sequences to the unaligned Pfam sequences and randomly shuffled the sequence order before making alignments and proceeding with the benchmark process as usual. We then calculated both the average ContTest score and the average sum-of-pairs score (SPS) for the HOMSTRAD sequences, thus arriving at two measures of quality for the same alignments.

### 2.7 Measures of guide tree imbalance

The Sackin score of a rooted, binary tree is defined as the sum of the depths of its leaves (Sackin, 1972). The Sackin score for a tree with *N* leaves, that is as balanced as possible is $N*{\text{log}}_{\text{2}}(N)$; the Sackin score for a perfectly imbalanced (chained) tree with *N* leaves is (N+2)*(N−1)/2. Concrete values for trees of up to eight leaves can be found in Sievers *et al.* (2014).

The expected value of the Sackin score depends on the underlying evolutionary model. In the Equal Rates Markov or Yule (Yule *etal.*, 1925) model, trees are built up, beginning with a node with just two leaves, by repeatedly selecting a leaf at random and replacing it with a node with two leaves until the required number of leaves is reached. The expected Sackin score for a tree with N leaves is calculated under the Equal Rates Markov model as:

$$EY[S(N)]=2N\sum_{i=1}^{N}1/i$$

In contrast, under the uniform or Proportional to Distinguishable Arrangements model, all trees with the same number of leaves are assumed to be equally likely. This is not strictly a model of evolution but merely of tree growth. The expected Sackin score under the uniform or Proportional to Distinguishable Arrangements model *EU*[*S*(*N*)] can be calculated as a hypergeometric function 3F2 (Mir *etal.*, 2013), which for large values of *N* is asymptotic to $\sqrt{\pi }N^{3/2}$.

## 3 Results

We developed ContTest, a benchmark for large protein MSAs based on the accuracy of *de novo* contact map prediction. ContTest contains 136 test cases with between 1467 and 43 910 sequences (median 7098) and an average length of between 31.9 and 370 residues (median 113.6) (see Supplementary Table S1 and Supplementary Data). We used the benchmark to score alignments made by Clustal Omega (Sievers *etal.*, 2011), Kalign 2 (Lassmann *etal.*, 2009), MAFFT (Katoh and Standley, 2013), MUSCLE (Edgar, 2004), Clustal W2 (Larkin *etal.*, 2007), Kalign 1 (Lassmann and Sonnhammer, 2005) and hmmt (Eddy, 1998). Additionally, the Pfam database contains MSAs of all sequences in each protein family, and we used the benchmark to score the full alignments from Pfam 27. Clustal Omega, Kalign 2, MAFFT and hmmt were all run with default parameters. To make alignments in a reasonable time, Kalign 1 was run with the fast heuristic -f option, MUSCLE was limited to two iterations and Clustal W2 reused the guide tree generated by Clustal Omega. Benchmark scores for these alignment methods are listed in Table 1.

**Table 1.** Benchmark scores for alignments from Pfam and seven MSA packages

Alignment method	Mean PSICOV precision	Mean EVfold-mfDCA precision	ContTest score	
hmmt	0.526	0.590	0.551	
				〉NS
Kalign1 (fast)	0.527	0.581	0.550	
				〉NS
Pfam	0.535	0.577	0.545	
				〉 **
Kalign 2	0.507	0.533	0.513	
				〉 ***
Clustal Omega	0.497	0.390	0.428	
				〉 **
MAFFT (default)	0.433	0.379	0.396	
				〉NS
Clustal W2	0.413	0.373	0.381	
				〉NS
MUSCLE (2 iterations)	0.445	0.329	0.371	

Mean PSICOV precision and mean EVfold-mfDCA precision are the mean precisions of the top L/5 long range contacts predicted by PSICOV and EVfold-mfDCA, respectively. ContTest Score is the geometric mean of the PSICOV and Evfold-mfDCA precisions. Statistical significances are indicated for the differences in consecutive pairs of ContTest scores. *P < 0.007; **P < 0.0014; ***P < 0.00014 (0.05, 0.001 and 0.001 with Bonferroni correction for seven tests, respectively); NS, not significant.

At the top of the rankings are the hmmt, Kalign 1 and Pfam alignments. These results are striking and unexpected. Since the method by which the Pfam alignments are created is not directly applicable to general MSA problems, the quality of Pfam alignments has not previously been assessed in MSA benchmark studies. Similarly, neither Kalign 1 nor hmmt have, to our knowledge, been considered in any recent benchmarking studies, as Kalign 1 is superseded by Kalign 2 and hmmt is not included in recent versions of HMMER.

The Pfam alignments are created in two steps. First, a manually curated ‘seed’ alignment is created from a small set of sequences representative of the sequence family. This seed alignment is used to train an HMM which is then used to search a sequence database and align matching sequences to the seed alignment. We note that this process—aligning sequence matches from a database one-by-one to a small seed alignment—is effectively performing progressive alignment with an almost perfectly chained guide tree. Kalign 1 uses a traditional progressive MSA strategy, with pairwise sequence distances estimated by approximate k-tuple matching. However, we found that Kalign 1 with the fast ‘-f’ option skips pairwise distance calculations and performs alignment with a fully chained guide tree, although this is an undocumented feature. Meanwhile, hmmt produces an MSA as a side effect of training an HMM on the input sequences and does not use a guide tree as part of this process. Thus, the sequence-clustering step which is characteristic of progressive MSA is not performed by any of these alignment methods.

**Fig. 2.**
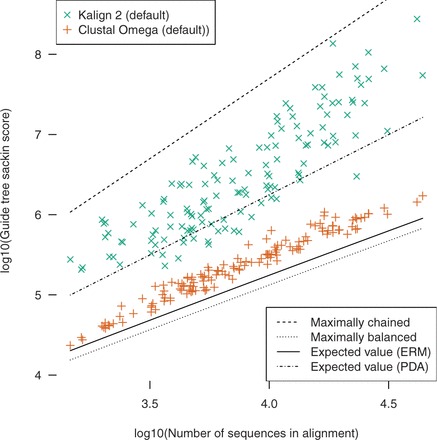
Comparison of Kalign 2 and Clustal Omega guide tree imbalance. The Sackin score (sum of distances from leaves to root) produced by each program is plotted against the number of sequences in the alignment for each test case. Values for fully chained and balanced trees and expected values under the Equal Rates Markov and Proportional to Distinguishable Arrangements models of tree growth are indicated with lines

The next highest scoring method is Kalign 2 (Table 1). Kalign version 2, like version 1, is a progressive aligner but lacks the option of its predecessor to use a fully chained guide tree. However, it uses the Muth-Manber algorithm to perform approximate k-tuple matching to estimate pairwise sequence similarities before constructing the guide tree. The pairwise distance metric used tends to produce highly chained guide trees (Boyce *etal.*, 2014). We find that this is true for our test cases, with Kalign 2 guide trees having high measures of imbalance when compared with Clustal Omega (Fig. 2), MAFFT or MUSCLE (Supplementary Figs S2 and Supplementary Data). This appears to be related to the length of the sequences aligned, with shorter average sequence lengths resulting in more chained guide trees (Supplementary Fig. S4).

**Fig. 3.**
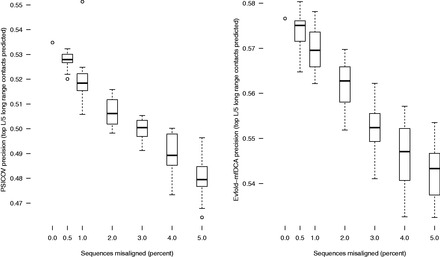
Introducing random misalignments decreases the benchmark score. Each boxplot represents 20 replicates where a different random subset of sequences is misaligned. There is a strong correlation between more errors and decreasing benchmark score

### 3.1 Varying alignment parameters

To verify that the ContTest score correctly ranks alignment methods by accuracy, we created alignments using different parameter sets of the same MSA packages. For a given alignment package, we expect that certain parameter sets produce more accurate alignments than others, on average. These relationships are reflected in the benchmark scores (Table 2).

**Table 2.** Benchmark scores for a variety of parameter sets of MAFFT, MUSCLE and Clustal Omega

Alignment method	Mean PSICOV precision	Mean EVfold-mfDCA precision	ContTest score
Clustal Omega (chained)	0.501	0.540	0.513***
Clustal Omega (Pfam HMM)	0.527	0.420	0.459***
Clustal Omega (3 iterations)	0.510	0.400	0.441 NS
Clustal Omega (2 iterations)	0.516	0.398	0.441 NS
Clustal Omega (default)	0.497	0.390	0.428

MAFFT (chained)	0.509	0.530	0.517***
MAFFT (default)	0.433	0.379	0.396
MAFFT NW-NS-PartTree-1	0.469	0.334	0.389 NS

MUSCLE (chained, 2 iterations)	0.508	0.553	0.526***
MUSCLE (2 iterations)	0.445	0.329	0.371

MUSCLE (chained, 1 iteration)	0.499	0.541	0.516***
MUSCLE (1 iteration)	0.415	0.314	0.354

Statistical significances of the differences in ContTest score between default and variant parameters of each package is indicated. Clustal Omega with chained guide trees, external HMM and two and three iterations are compared with default Clustal Omega scores. MAFFT PartTree and MAFFT with chained guide trees are compared with default MAFFT. MUSCLE with two iterations and a starting chained guide tree is compared with two iterations of MUSCLE. MUSCLE with a chained guide tree and one iteration is compared with MUSCLE with one iteration.

***P < 0.001.

Both Clustal Omega and MUSCLE are capable of iteratively refining MSAs and using more iterations results in an increase in benchmark score for these methods (Table 2). In addition, Clustal Omega can use an external HMM to guide alignments. Using the Pfam HMMs for each family during alignment also increases the benchmark score for Clustal Omega. The NW-NS-PartTree-1 parameter set of MAFFT is recommended for making fast alignments of large numbers of sequences and is expected to have a lower average accuracy than the default parameters. We observe a corresponding decrease in the benchmark score for MAFFT when using these parameters.

As noted above, the top three highest scoring sets of alignments, those from hmmt, Kalign 1 (fast) and Pfam, were created without the use of a conventional guide tree. To determine the extent to which the lack of sequence clustering contributes to these methods having the highest scoring alignments, we created alignments using MAFFT, MUSCLE and Clustal Omega with fully chained guide trees (Table 2). Kalign 2 does not accept external guide trees, while Clustal W ran prohibitively slowly when using chained guide trees. In all cases, alignments made using chained guide trees score higher on average than alignments made with the guide trees constructed internally with each package. With MUSCLE and Clustal Omega, we see that the score increase due to chained guide trees is greater than that due to any of the other alternative parameters tested.

### 3.2 Adding errors to alignments decreases the benchmark score

As a further test of benchmark accuracy, we studied how the addition of deliberate errors to a set of alignments affects the benchmark score. First, we selected random subsets of sequences in each Pfam alignment to ‘misalign’ by adding a gap to the start of the aligned sequence, while truncating the final character (see Section 2.5). We observed that greater the percentage of sequences in each alignment that were misaligned, the greater the drop in contract prediction precision from the starting alignments. Figure 3 shows PSICOV and EVfold-mfDCA precisions for alignments with 0.5%, 1%, 2%, 3%, 4% and 5% misaligned sequences. Second, we performed a similar analysis by randomly mutating 5%, 10%, 15% or 20% of individual residues in each alignment. We found a clear correlation between increasing mutations and decreasing contact prediction precision (Supplementary Fig. S6).

### 3.3 Adding HOMSTRAD sequences to alignments allows a second measurement for alignment quality

To compare the ContTest benchmark method with the more traditional structure-superposition benchmark method, we created a hybrid benchmark by adding HOMSTRAD reference sequences to the Pfam sequences in 80 test cases for which we identified a match between the Pfam family and a HOMSTRAD alignment. We then calculated both the ContTest score of each alignment and the SPS for the embedded HOMSTRAD sequences. Scores for the HOMSTRAD sequences are listed in Table 3. A plot of the ContTest score against the mean HOMSTRAD SPS is shown in Supplementary Figure S7. The SPS and the ContTest scores notably disagree on Clustal Omega, which is the most accurate method as ranked by SPS. MAFFT NW-NS-PartTree-1 and Kalign 2 also differ slightly in their rankings between the two benchmark scores. It is unclear why Clustal Omega is ranked so differently by the two accuracy measures. Since the SPS measures only the number of correctly aligned residue pairs in an alignment, it does not penalize ‘overalignment’, i.e. false-positive aligned residues in the MSA. It is possible that MSA methods which are tuned for sensitivity over specificity may be ranked higher when measured by SPS or total column score than when ranked by ContTest or other accuracy measures.

**Table 3.** Sum-of-pairs scores and ContTest scores for 80 test cases with embedded HOMSTRAD sequences

Alignment method	Mean SP score	ContTest score
Clustal Omega (default)	0.722	0.410
Kalign 1 (fast)	0.722	0.548
hmmt	0.718	0.543
Kalign 2	0.709	0.507
MUSCLE (2 iterations, chained)	0.696	0.519
MUSCLE (1 iteration, chained)	0.676	0.509
MAFFT FFT-NS-2	0.610	0.376
MUSCLE (2 iterations)	0.588	0.355
MAFFT NW-NS-PartTree-1	0.567	0.389
MUSCLE (1 iteration)	0.529	0.331

### 3.4 ContTest rankings differ from PREFAB rankings

PREFAB (Edgar, 2004) is a structure-superposition-based MSA benchmark containing 1682 test cases. Test cases each contain two sequences for which a reference alignment is available and up to 48 other homologs. We calculated the SPS on the PREFAB benchmark for all MSA methods for which we previously calculated ContTest scores (Supplementary Table S4). The rankings of alignment methods differ greatly between ContTest and PREFAB, with hmmt and ‘chained’ alignments scoring particularly poorly on PREFAB, while being among the highest scoring methods when ranked by ContTest. The difference in ranking is unsurprising given the large difference in numbers of sequences between PREFAB and ContTest. ‘Chained’ guide trees have previously been shown to be effective only for alignments of large numbers of sequences (Boyce *etal.*, 2014). Meanwhile, the greater sequence diversity present in the larger ContTest test cases is likely to facilitate the HMM training at the core of hmmt’s alignment process. It should be noted that for alignments of tens of sequences, as in PREFAB, many slower, more accurate MSA methods are available which cannot make MSAs of the size required for the ContTest benchmark and thus were not included in our study.

## 4 Discussion

ContTest is the first protein MSA benchmark to realistically test alignments of large numbers of sequences and base scores on empirical biological data. Although structure-based benchmarks may contain alignments with many sequences, e.g. HomFam (Blackshields *etal.*, 2010), scores are always based on reference alignment of a small subset of sequences contained in the MSA. This could allow large errors in non-reference sequences to go unpunished. In addition, the structure-based reference alignment must itself be created, and this alignment depends on heuristic structure comparison algorithms. Meanwhile, the phylogeny-based species-discordance benchmark of Dessimoz and Gil (2010)—in which phylogenetic trees are inferred from MSAs and compared with a known reference tree—is restricted to testing alignments of six sequences. Simulation-based benchmarks allow for arbitrary numbers of sequences in test alignments but depend on simplified models of evolution and may not realistically model gap placement in real proteins. Our benchmark method uses information from all sequences in the input alignments. Test cases must contain a minimum of about 1000 sequences, so there is sufficient information in the alignment to make contact predictions, but there is no practical upper limit on the number of sequences.

Our benchmark scores confirm the utility of completely chained guide trees, as described by Boyce *etal.* (2014). That result has been controversial (Tan *etal.*, 2015) but, at least for alignments of many sequences from structurally conserved regions, the results appear clear and robust. Alignments made with fully or even largely chained guide trees consistently outscore alignments made with more traditional guide trees. Each of the alignment packages that accepts user-specified guide trees benefitted from using chained guide trees, and the increase in score due to chained guide trees was greater than the increase due to extra iterations of Clustal Omega or MUSCLE or using a high quality external HMM with Clustal Omega. Our scores also demonstrate the quality of the alignments in the Pfam database, which may have previously been neglected, as well as suggesting that both Kalign 1 and hmmt be revisited as useful methods for creating alignments of large numbers of sequences.

We have shown that the ContTest benchmark is in general agreement with structure-based benchmarks for large numbers of sequences. However, we observe that the methods that produce the most accurate alignments for large numbers of sequence do not necessarily perform well for small number of sequences, as measured by the PREFAB benchmark (Supplementary Table S4). ContTest is likely to be most sensitive to alignment errors in regions which are structurally conserved but without very high levels of sequence conservation. That is, regions of the protein in which amino acid substitutions are accepted but must be compensated for at co-evolving sites. A limitation of this benchmark is that all alignments inherently involve only sequences of a single protein domain, without any large insertions. It is likely that certain methods might perform poorly on more general alignment problems involving multiple domains. In particular, for hmmt alignments, it is necessary that a sensible HMM can be trained on the input sequences, which may not be possible in the general case. However, these are problems that may be addressed by future benchmarks, and alignments of single protein domains are common and important in many applications.

Finally, these results give some clear pointers toward the best methods for making MSAs intended for use with *de novo* structure prediction methods and show that choice of alignment method can have a large influence on the quality of contact predictions, even when using the same software package with different parameters.

## Funding

This work was supported by Science Foundation Ireland through PI grant 11/PI/1034 (to D.G.H.).

*Conflict of Interest*: none declared.

## Supplementary Material

Supplementary Data
