# Supplementary material for: Using de novo protein structure predictions to measure the quality of very large multiple sequence alignments
Source: Bioinformatics. 2015 Nov 14;32(6):814–20. doi: 10.1093/bioinformatics/btv592 (PMC5939968; doi:10.1093/bioinformatics/btv592)
Supplement: Supplementary Data [file btv592_supplementary_data.zip › supplement.pdf]

SUPPLEMENTARY INFORMATION: Using De Novo Protein Structure Predictions to Measure The Quality Of Very Large Multiple Sequence Alignments

Gearóid Fox, Fabian Sievers, and Desmond G Higgins

Conway Institute of Biomolecular and Biomedical Research and UCD School of Medicine and Medical Science, University College Dublin, Dublin 4, Ireland

Supplementary Table 1: Benchmark Test Cases. Pfam ID is the ID of the Pfam family supplying the sequences for each test case. UniProt ID is the identifier for the target protein for which contact predictions are made. PDB ID is the ID of the structure from the Protein Data Bank supplying 3D coordinates for the target protein. Sequences and Average Length are the number of sequences in each alignment and the average length of the unaligned sequences, respectively.

Pfam Id	Protein ID	PDB ID	Sequences	Average Length
PF00012	HSP7F_CAEEL	3dqgA	23002	370.0
PF00014	A4_HUMAN	1aapA	4915	53.4
PF00017	GRB10_HUMAN	1nrvA	9081	78.0
PF00018	ABP1_YEAST	1jo8A	10749	47.1
PF00025	SAR1B_CRIGR	1f6bA	5460	161.8
PF00027	CNGK1_RHILO	1vp6A	26013	90.2
PF00030	DESS_MYXXA	1npsA	3340	81.1
PF00031	CYTD_HUMAN	1roaA	2054	88.6
PF00034	CY552_THETH	1c52A	12531	94.0
PF00042	MYG_PHYMC	1a6mA	6000	99.8
PF00045	HEMO_RABIT	1hxnA	5321	44.7
PF00048	CCL20_HUMAN	1m8aA	2117	63.8
PF00051	APOA_HUMAN	1i71A	2697	76.9
PF00059	OC17_CHICK	1gz2A	12227	107.5
PF00061	LACB_BOVIN	1bebA	2221	128.6
PF00071	VPS21_YEAST	1ek0A	21243	151.9
PF00075	RNH_ECOLI	1jl1A	16967	121.4
PF00080	CCS1_YEAST	1ej8A	3852	143.2
PF00085	Q9NG23_TRYBB	1r26A	24231	102.3
PF00089	TRY1_BOVIN	5ptpA	22248	206.2
PF00092	VWF_HUMAN	1atzA	10632	166.7
PF00104	RARG_HUMAN	1fcyA	7507	187.9
PF00111	FER1_SPIOL	1a70A	17474	75.2
PF00112	ERVB_TABDI	1iwdA	7017	199.9
PF00116	COX2_THETH	2cuaA	31269	106.0
PF00118	CH60_ECOLI	1kidA	23232	316.1
PF00121	TPIS_LEIME	2vxnA	7470	212.1
PF00127	PLAS_SPIOL	1ag6A	1467	103.8
PF00139	LEC_ERYCG	1gzcA	1651	204.2
PF00144	BLAC_STRAL	1bsgA	13942	321.5
PF00160	CYP5_CAEEL	1h0pA	13237	159.3
PF00168	KPCE_RAT	1gmiA	23398	86.1
PF00169	PKHA1_HUMAN	1eazA	16347	107.3
PF00173	CYB5_ECTVA	1cxyA	5879	82.9
PF00186	DYR_CANAX	1aoeA	5237	156.6
PF00211	CY41_TRYBB	1fx2A	9831	176.3
PF00234	NLTP_MAIZE	1fk5A	1632	103.1
PF00237	RL22_THETH	1i4jA	6446	106.9
PF00238	RL14_GEOSE	1whiA	6114	119.8
PF00241	TWF1_MOUSE	1m4jA	2431	121.7
PF00254	MIP_TRYCR	1jvwA	16739	99.8
PF00258	FLAV_SYNE7	1cznA	10556	138.5
PF00293	AP4A_CAEEL	1ktgA	43912	130.9
PF00300	Q9ALU0_GEOSE	1h2eA	22415	153.4
PF00301	RUBR_PYRFU	1brfA	2943	46.1
PF00307	SPTB2_HUMAN	1bkrA	10780	104.8

Pfam Id	Protein ID	PDB ID	Sequences	Average Length
PF00313	CSPB_BACCL	1c9oA	14886	65.2
PF00355	BPHF_BURXL	1fqtA	13057	94.2
PF00359	PTM3C_ECOLI	1a3aA	18510	141.3
PF00364	BCCP_ECOLI	1bdoA	25099	71.8
PF00381	PTHP_MYCCT	1pchA	8181	83.3
PF00403	ATX1_YEAST	1cc8A	19664	61.2
PF00406	KCY_DICDI	1qf9A	9734	160.6
PF00413	MMP1_HUMAN	1hfcA	3213	160.9
PF00445	RNLE_SOLLC	1dixA	3203	162.7
PF00462	GLRX_BPT4	1abaA	11246	62.3
PF00515	NCF2_HUMAN	1hh8A	18473	31.9
PF00542	RL7_ECOLI	1ctfA	5164	67.2
PF00550	ACP_ECOLI	1t8kA	41592	66.4
PF00551	PUR3_ECOLI	1jkxA	13471	174.1
PF00565	NUC_STAAU	1ihzA	4232	105.4
PF00572	RL13_PYRHO	1j3aA	5540	124.9
PF00573	RL4_THEMA	1dmgA	5579	191.4
PF00581	GLPE_ECOLI	1gmxA	30295	101.9
PF00595	NHRF1_HUMAN	1g9oA	26099	80.3
PF00626	SEVE_DICDI	1svyA	5387	78.0
PF00652	MRC1_MOUSE	1dqgA	3906	120.8
PF00657	RHA1_ASPAC	1k7cA	4517	269.3
PF00787	NCF1_HUMAN	1kq6A	7076	114.9
PF00857	O58727_PYRHO	1im5A	13319	172.1
PF00929	ERI1_HUMAN	1w0hA	17634	160.3
PF00959	LYS_BPT4	1lpyA	3255	110.6
PF00989	PYP_HALHA	2phyA	15580	102.2
PF01029	NUSB_THEMA	1tzvA	7994	125.8
PF01135	PIMT_HUMAN	1i1nA	3573	199.2
PF01152	TRHBN_PARCA	1dlwA	2140	116.8
PF01161	PEBP1_HUMAN	1behA	4705	145.5
PF01183	LYSM1_STRGL	1jfxA	3628	177.3
PF01195	CRS2_MAIZE	1rybA	4825	180.0
PF01223	NUCA_SERMA	1ql0A	2367	199.7
PF01250	RS6_THEMA	1vmbA	4748	91.7
PF01300	YCIO_ECOLI	1k7jA	8425	175.0
PF01323	DSBA_ECOLI	1fvkA	5572	172.9
PF01327	DEF_STAAU	1lm4A	7120	154.6
PF01339	CHEB_SALTY	1chdA	3655	180.3
PF01363	VPS27_YEAST	1vfyA	4289	71.1
PF01421	VM1AD_CROAT	1atlA	3201	190.2
PF01451	PPAL_YEAST	1d1qA	7884	133.1
PF01464	LYG_CYGAT	1gbsA	14235	115.8
PF01520	Q9LCR3_PAEPO	1jwqA	7380	194.9
PF01522	PDAA_BACSU	1ny1A	13288	133.5
PF01541	TEV1_BPT4	1mk0A	9798	77.8
PF01588	AIMP1_HUMAN	1fl0A	10729	99.1
PF01625	MSRA_BOVIN	1fvgA	6530	153.9
PF01627	CHEA_THEMA	1tqgA	11727	93.1
PF01661	Y1521_ARCFU	1vhuA	5297	112.3
PF01668	SSRP_THET8	1wjxA	4544	67.8
PF01687	RIFK_HUMAN	1nb9A	4738	126.9
PF01728	RLME_ECOLI	1ej0A	10200	173.8
PF01740	SP2AA_LYSSH	1h4xA	10190	111.4
PF01807	PRIM_GEOSE	1d0qA	5345	96.1

Pfam Id	Protein ID	PDB ID	Sequences	Average Length
PF01812	Q5SHW9_THET8	1wkcA	4810	177.4
PF01814	HEMTM_THEHE	2mhrA	6352	127.9
PF01965	HSP31_YEAST	1rw7A	9251	150.2
PF01985	Y1333_HAEIN	1jo0A	3330	83.8
PF02033	RBFA_HAEIN	1josA	4543	105.0
PF02036	NLTP_RABIT	1c44A	3603	100.0
PF02214	KCNA2_RAT	1dsxA	3855	92.0
PF02224	KCY_ECOLI	1ckeA	4159	155.7
PF02302	PTQB_ECOLI	1iibA	18949	89.1
PF02311	ARAC_ECOLI	2arcA	10833	132.6
PF02367	Y065_HAEIN	1htwA	4392	122.7
PF02527	RSMG_BACSU	1xdzA	4375	182.2
PF02579	Q9F5X9_AZOVI	1p90A	2027	94.2
PF02581	THIE_BACSU	2tpsA	5722	177.9
PF02597	Q8U3C7_PYRFU	1vjkA	6781	72.9
PF02861	CLPA_ECOLI	1k6kA	16656	51.2
PF03009	Q9X1V6_THEMA	1o1zA	9993	235.0
PF03061	4HBT_PSEUC	1lo7A	18406	79.3
PF03167	MUG_ECOLI	1mugA	10115	160.0
PF03960	Q9HXX5_PSEAE	1rw1A	7591	107.4
PF04073	YBAK_HAEIN	1dbxA	9872	122.0
PF04296	Q97S59_STRPN	1g2rA	2403	77.8
PF04509	CHEC_THEMA	1xkrA	1993	37.7
PF04545	Q9EZJ8_THEAQ	1ku3A	17028	51.6
PF05198	IF3_GEOSE	1tifA	4308	67.6
PF06325	Q9HIL9_THEAC	1ne2A	4673	251.4
PF06445	SBMC_ECOLI	1jyhA	6066	155.4
PF07647	PHP_DROME	1kw4A	3625	64.9
PF07653	MIA_HUMAN	1i1jA	4617	59.2
PF07686	MOG_RAT	1pkoA	19537	107.8
PF08534	TLPA_BRAJA	1jfuA	9684	140.6
PF13460	BLVRB_HUMAN	1hdoA	14495	186.9
PF13499	TNNC2_CHICK	1avsA	22951	70.2
PF13905	O77093_CRIFA	1i5gA	2061	94.5
PF13921	MYB_MOUSE	1guuA	4769	61.9

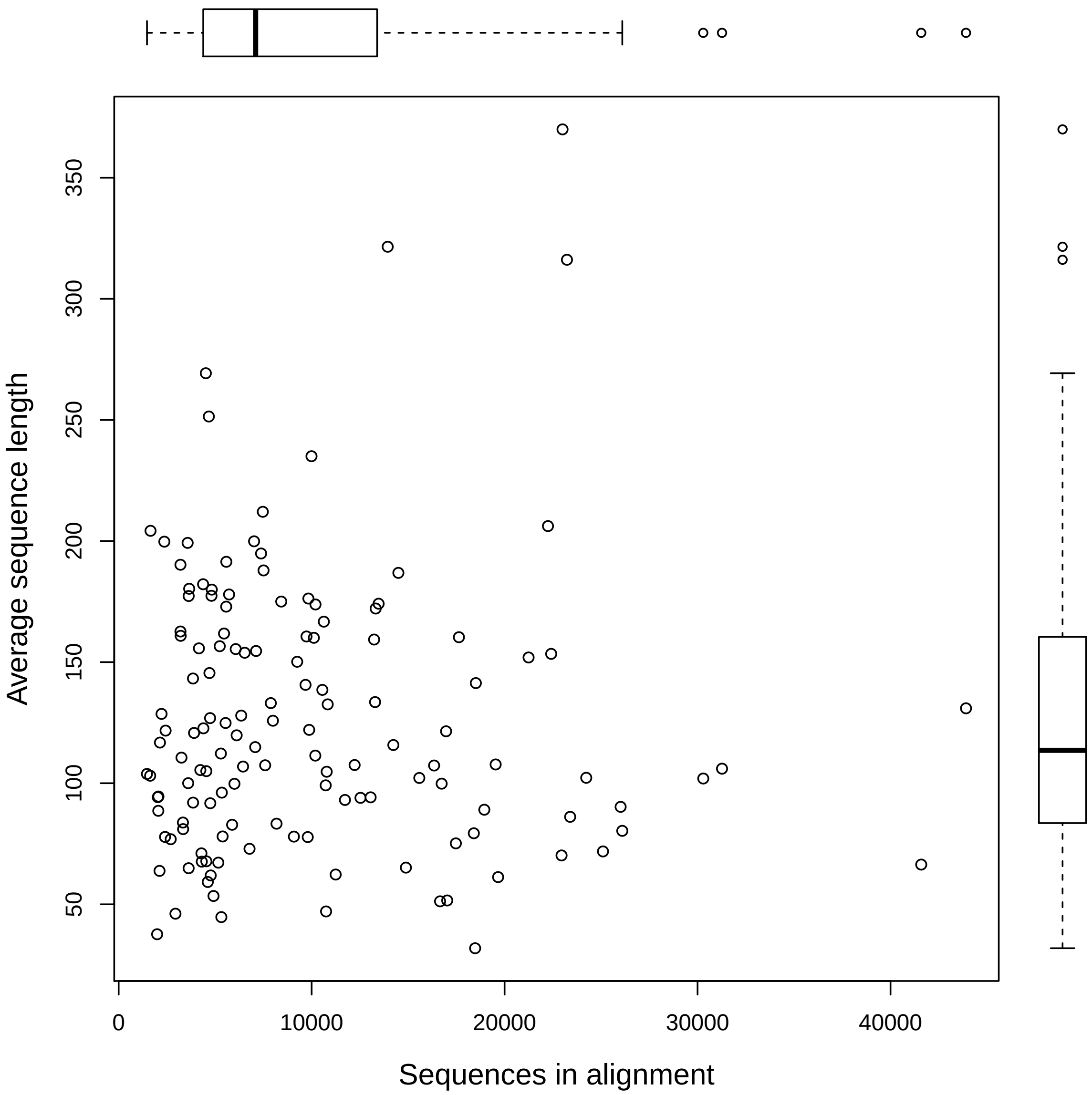

Supplementary Figure 1: Test case dimensions. Average sequence length in each of the 136 test cases is plotted against the number of sequences in that test case.

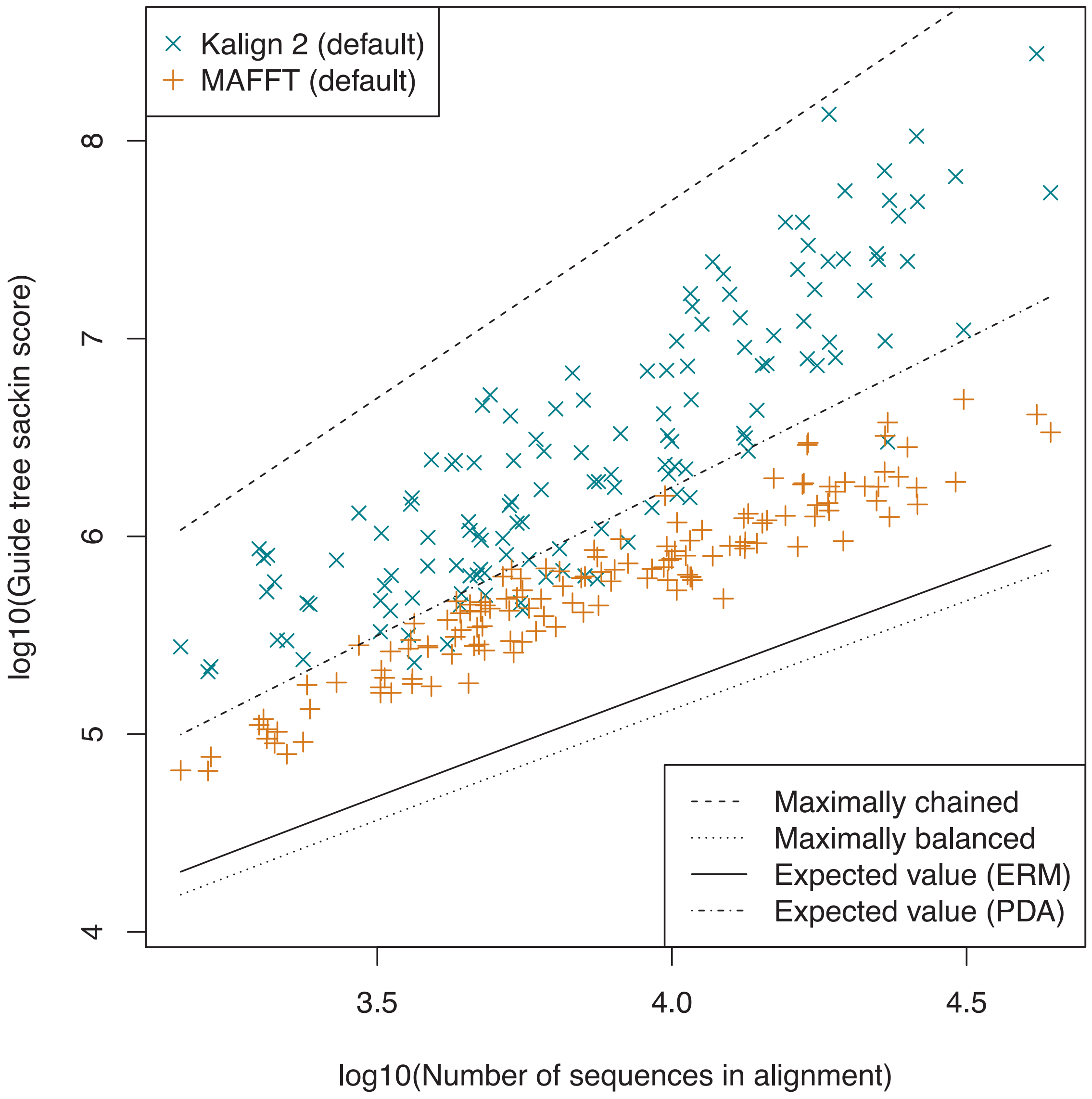

Supplementary Figure 2: Comparison of Kalign 2 and MAFFT guide tree imbalance. The Sackin score (sum of distances from leaves to root) of the default guide tree produced by each program is plotted against the number of sequences in the alignment for each test case. Values for fully chained and balanced trees, and expected values under two models of tree growth are indicated with lines.

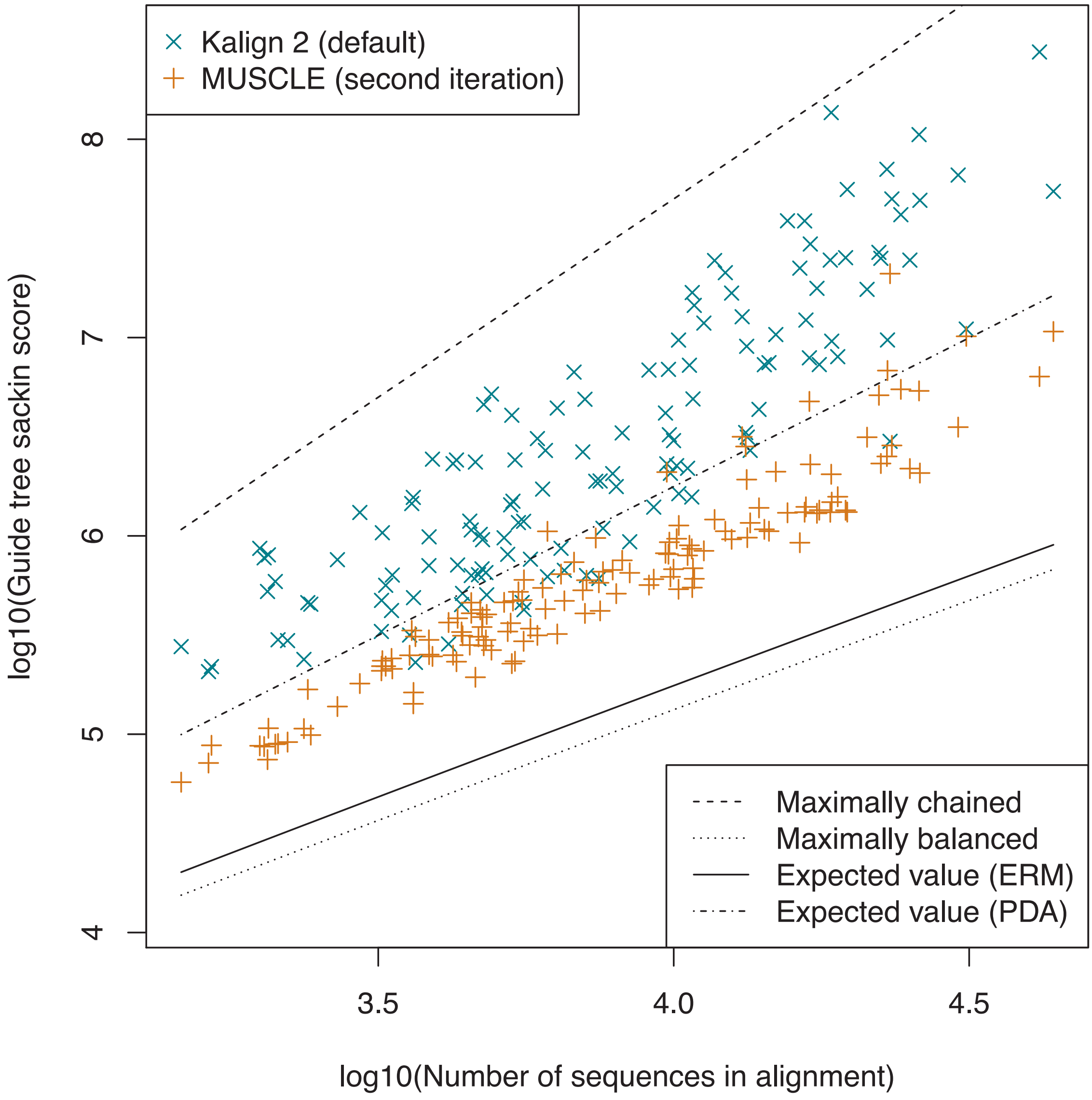

Supplementary Figure 3: Comparison of Kalign 2 and MUSCLE guide tree imbalance. The Sackin score (sum of distances from leaves to root) of the default guide tree produced by each program is plotted against the number of sequences in the alignment for each test case. Values for fully chained and balanced trees, and expected values under two models of tree growth are indicated with lines.

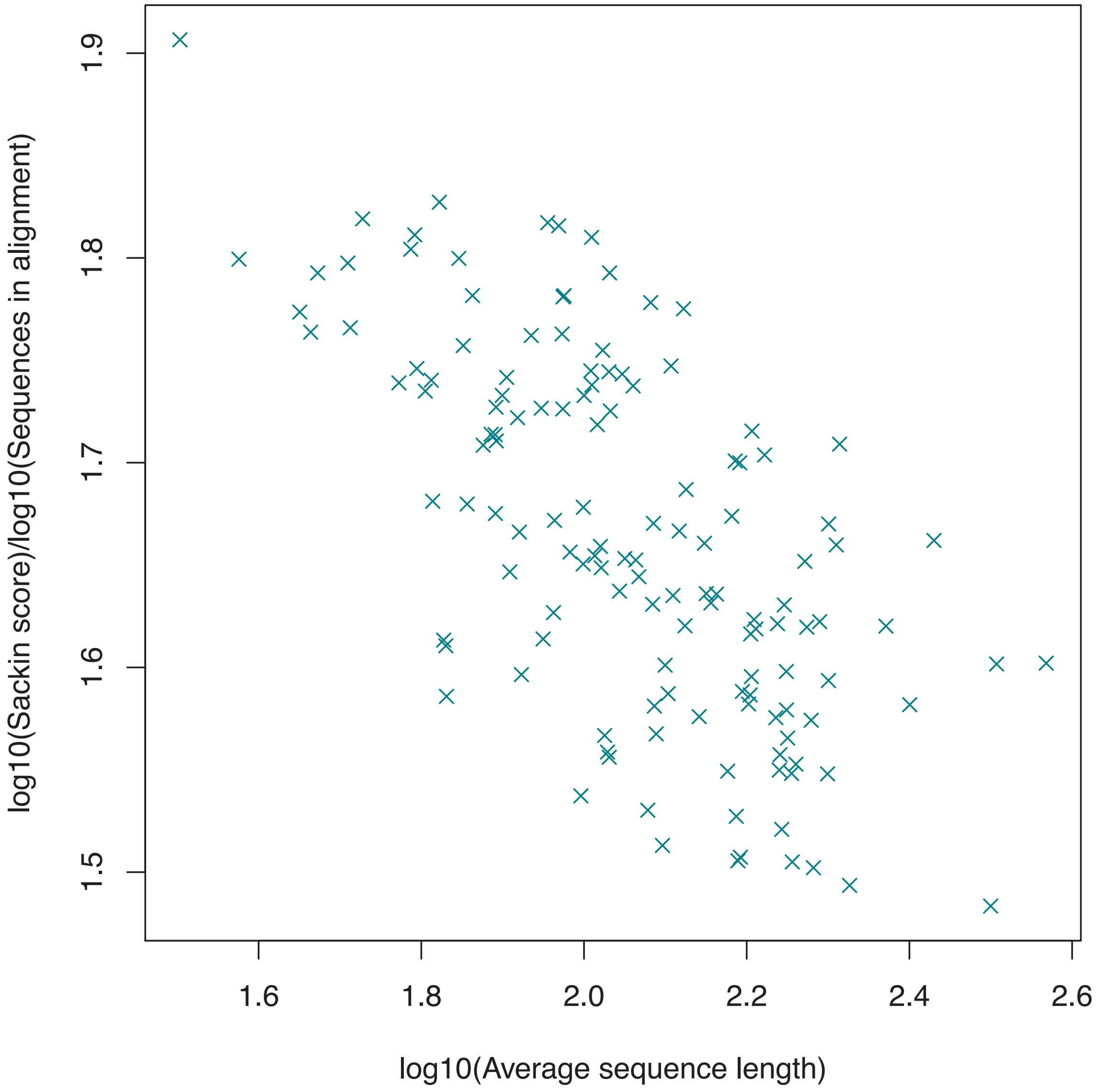

Supplementary Figure 4: The degree of Kalign 2 guide tree imbalance is proportional to average sequence length. The Sackin score of the default Kalign 2 guide tree, normalised by the total number of sequences in the alignment, is plotted against the average sequence length for each test case. Shorter sequences tend to result in more imbalanced guide trees.

Supplementary Text: Using ROC curves to measure contact prediction accuracy

We use the precision of the top L/5 contacts to measure the accuracy of predicted contacts and formulate our benchmark score. However, this necessarily involves only a subset of the columns in an alignment in the final score for the alignment. As an alternative, we can use Receiver Operating Characteristic (ROC) curves to measure the accuracy of contact prediction. This has the advantage of including information from all columns in the alignment in the final score. We show here that the choice of scoring method has little effect on the final rankings of MSA methods.

EVfold-mfDCA and PSICOV output lists of predicted contacts ranked by a confidence score. Predicted contacts with a confidence score above a certain threshold can be compared to the list of reference contacts and true positive and false positive predictions can be counted. The ROC curve is the plot of the true positive rate against the false positive rate of the predicted contacts as the threshold confidence score is varied. The area under this curve (AUC) is a measure of the accuracy of the contact predictions independent of the threshold confidence score. We can scale the AUC onto the interval 0–100 and use this as an alternative score for an alignment (Supplementary Figure 5). We will refer to this alternative score as the ROC-score.

To calculate the ROC-score, contact predictions are made by FreeContact and PSICOV 2, as described in the main text. Contacts between residues separated by 4 or fewer other residues are excluded, as PSICOV does not predict these contacts. That is, for residues i annd j, contacts are only considered where $|i - j| > 4$. The AUC of the ROC curve is calculated using the ROCR package for R (Carslon *et al.*, n.d.).

Using this scoring system, we arrive at the results shown in Supplementary Tables 2 and 3. We note two differences in the rankings between the ContTest score based on the top L/5 contacts and the alternative ROC-scores: Pfam alignments are the sole best alignments based on the ROC-scores, while 2 iterations of MUSCLE is ranked higher than MAFFT and Clustal W2. The effect of chained guide trees remains clear and significant regardless of which scoring method is used.

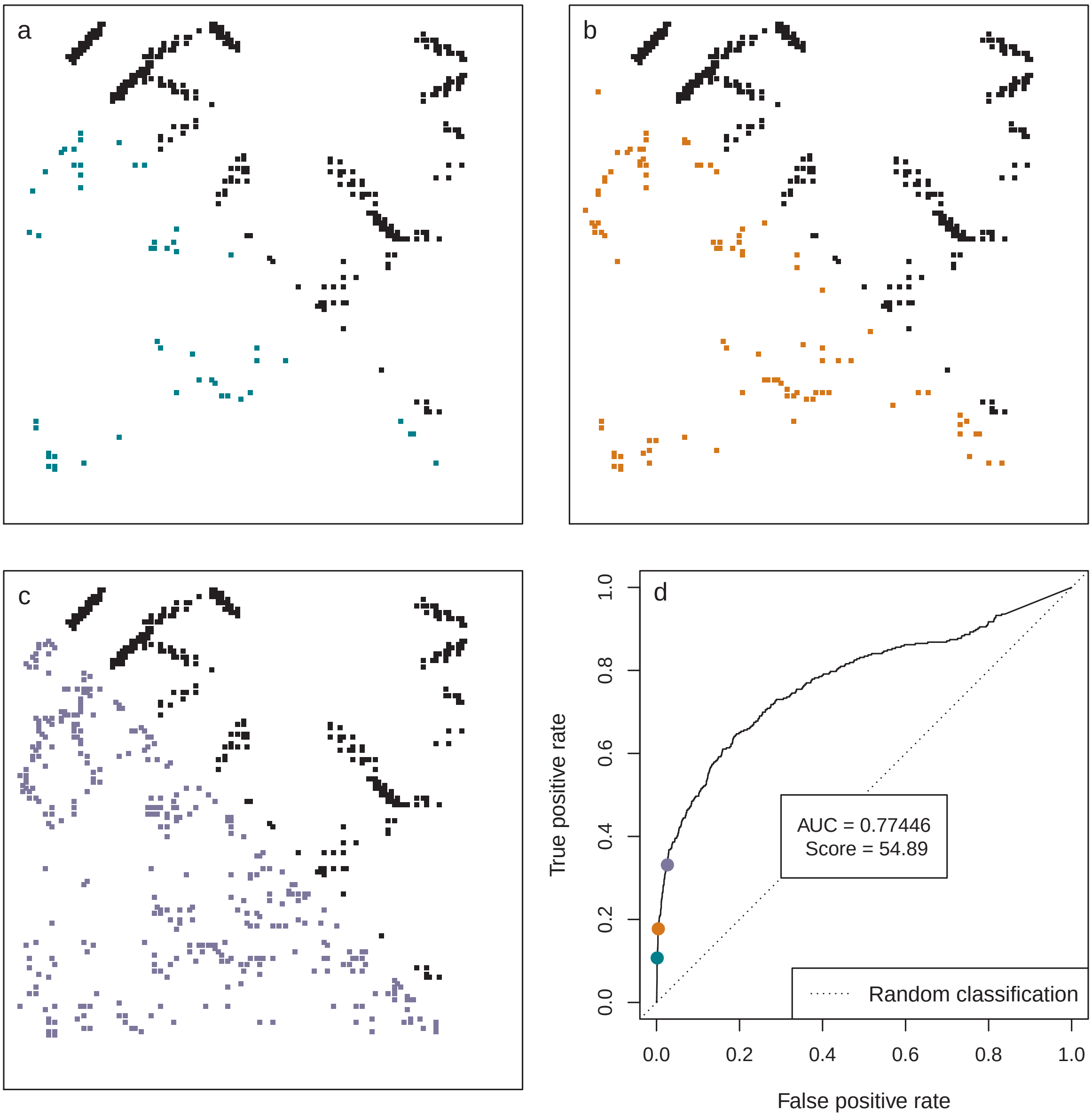

Supplementary Figure 5: Scoring predicted contacts. Contacts predicted for RNH_ECOLI from alignment of Pfam family PF00075 and compared to structure 1JL1. **(a—c)** predicted contacts at different score thresholds with true contacts. The upper right of each contact map shows contacts calculated from 1JL1 at 8Å C_β–C_β distance threshold. The lower left of each contact map shows contacts predicted from the PF00075 alignment using PSICOV 2 with a score cutoff of 1.0 **(a)**, 2.0 **(b)** and 3.0 **(c)**, respectively. **(d)** the ROC curve is found by plotting the TPR against the FPR of as the score threshold of the predicted contacts in varied. Points corresponding to the predicted contact maps above are highlighted. The score is calculated from the are under this curve.

Alignment Method	PSICOV ROC-score	EVfold-mfDCA ROC-score	ContTest ROC-score	
Pfam	42.83	54.36	48.60	$\rangle *$
Kalign1 (fast)	43.19	52.39	47.35	$\rangle NS$
hmmt	41.75	51.91	45.36	$\rangle NS$
Kalign 2	40.54	50.79	44.72	$\rangle * * *$
Clustal Omega	38.40	44.69	40.58	$\rangle *$
MUSCLE (2 iterations)	36.51	43.16	39.24	$\rangle NS$
MAFFT (default)	35.76	42.31	37.96	$\rangle **$
Clustal W2	31.91	42.15	35.56	

Supplementary Table 2: ROC-scores for alignments from Pfam and 7 MSA packages. PSICOV Score is the average score when using PSICOV to predict contacts from MSAs. EVfold-mfDCA Score is the average score when using FreeContact with the EVfold-mfDCA algorithm. ContTest Score is the geometric mean of the PSICOV and Evfold-mfDCA scores. Statistical significances are indicated for the differences in consecutive pairs of scores. $*$: $p < 0.007$; $**$: $p < 0.0014$; $* * *$: $p < 0.00014$ (0.05, 0.001, and 0.001 with Bonferroni correction for 7 tests, respectively); NS not significant.

Alignment Method	PSICOV ROC-score	EVfold-mfDCA ROC-score	ContTest ROC-score
Clustal Omega (chained)	40.25	48.36	44.31∗∗∗
Clustal Omega (Pfam HMM)	39.80	47.45	43.08∗∗∗
Clustal Omega (3 iterations)	39.26	45.71	41.71
Clustal Omega (2 iterations)	38.97	46.19	41.69∗
Clustal Omega (default)	38.40	44.69	40.58
MAFFT (chained)	40.44	48.81	44.28∗∗∗
MAFFT (default)	35.76	42.31	37.96
MAFFT NW-NS-PartTree-1	34.76	38.58	35.96∗∗
MUSCLE (chained, 2 iterations)	41.35	51.48	45.93∗∗∗
MUSCLE (2 iterations)	36.51	43.16	39.24
MUSCLE (chained, 1 iteration)	40.30	48.99	44.10∗∗∗
MUSCLE (1 iteration)	32.27	37.17	34.21

Supplementary Table 3: Benchmark scores for a variety of parameter sets of MAFFT, MUSCLE and Clustal Omega. Statistical significances of the differences between default and variant parameters of each package is indicated. Clustal Omega with chained guide trees, external HMM and two and three iterations are compared to default Clustal Omega scores. MAFFT PartTree and MAFFT with chained guide trees are compared to default MAFFT. MUSCLE with two iterations and a starting chained guide tree is compared to two iterations of MUSCLE. MUSCLE with a chained guide tree and one iteration is compared to MUSCLE with one iteration. ∗∗∗: $p < 0.001$; ∗∗: $p < 0.01$. Additionally, the score for one iteration of MUSCLE is significantly different from the score for two iterations $p < 0.001$.

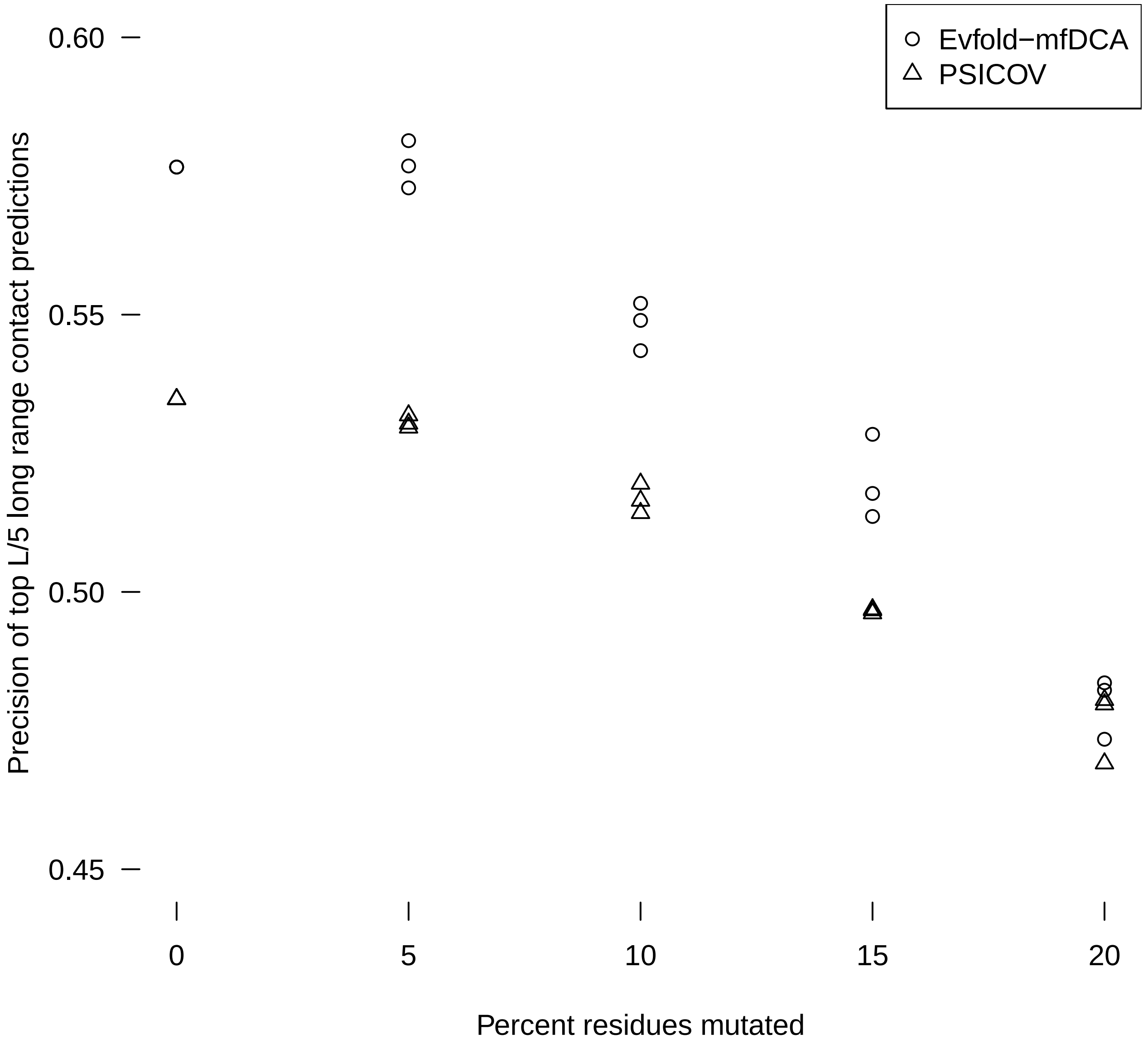

Supplementary Figure 6: Randomly mutating resiudes in a set of alignments reduces the precision of contact predictions.

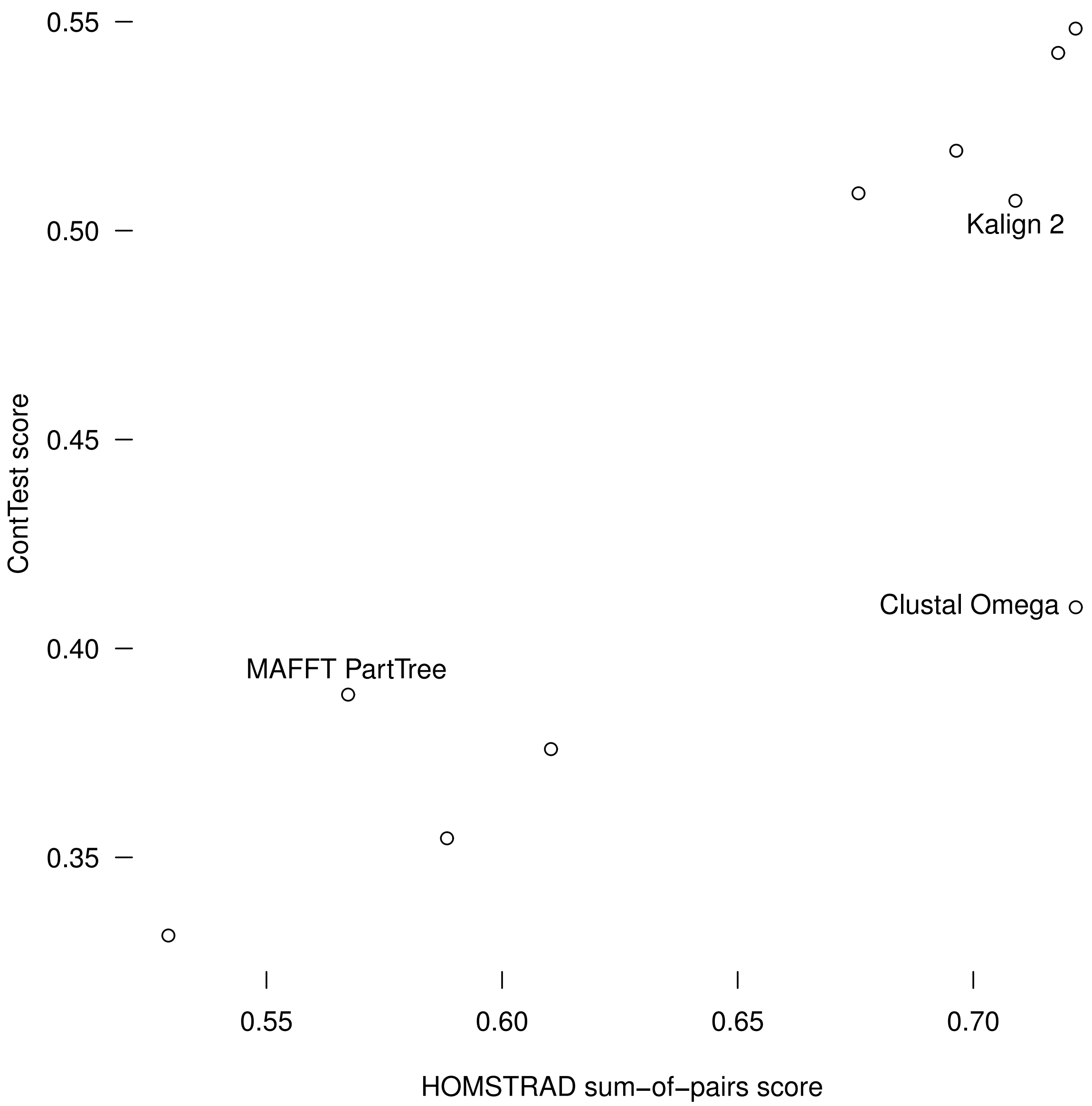

Supplementary Figure 7: Plot of ContTest score against mean sum-of-pairs score for 10 alignment methods on 80 test cases with embedded HOMSTRAD sequences. There is a positive correlation between the two scoring systems, although the Clustal Omega alignments are a notable outlier.

Alignment Method	Mean SP score
Clustal Omega	0.700
MAFFT	0.677
MUSCLE (2 iterations)	0.667
Kalign 2	0.649
MAFFT NW-NS-PartTree-1	0.643
Clustal Omega (chained)	0.640
MUSCLE (1 iteration)	0.639
MAFFT (chained)	0.629
MUSCLE (chained, 1 iteration)	0.619
Kalign 1 (fast)	0.618
Clustal W2	0.617
MUSCLE (chained, two iterations)	0.616
hmmt	0.476

Supplementary Table 4: Sum-of-pairs scores for alignment methods on the PREFAB benchmark.
